# Simulation Studies for Methodological Research in Psychology: A Standardized Template for Planning, Preregistration, and Reporting

**DOI:** 10.1037/met0000695

**Published:** 2024-11-14

**Authors:** Björn S. Siepe, František Bartoš, Tim P. Morris, Anne-Laure Boulesteix, Daniel W. Heck, Samuel Pawel

**Affiliations:** 1 Department of Psychology, University of Marburg; 2 Department of Psychological Methods, University of Amsterdam; 3 MRC Clinical Trials Unit at UCL, University College London; 4 Institute for Medical Information Processing, Biometry and Epidemiology, Faculty of Medicine, Ludwig-Maximilians-Universität München and Munich Center of Machine Learning; 5 Epidemiology, Biostatistics and Prevention Institute (EBPI) and Center for Reproducible Science (CRS), University of Zurich

**Keywords:** experimental design, Monte Carlo experiments, meta-research, preregistration, reporting

## Abstract

Simulation studies are widely used for evaluating the performance of statistical methods in psychology. However, the quality of simulation studies can vary widely in terms of their design, execution, and reporting. In order to assess the quality of typical simulation studies in psychology, we reviewed 321 articles published in *Psychological Methods, Behavior Research Methods*, and *Multivariate Behavioral Research* in 2021 and 2022, among which 100/321 = 31.2% report a simulation study. We find that many articles do not provide complete and transparent information about key aspects of the study, such as justifications for the number of simulation repetitions, Monte Carlo uncertainty estimates, or code and data to reproduce the simulation studies. To address this problem, we provide a summary of the ADEMP (Aims, Data-generating mechanism, Estimands and other targets, Methods, Performance measures) design and reporting framework from Morris, White, and Crowther (2019) adapted to simulation studies in psychology. Based on this framework, we provide ADEMP-PreReg, a step-by-step template for researchers to use when designing, potentially preregistering, and reporting their simulation studies. We give formulae for estimating common performance measures, their Monte Carlo standard errors, and for calculating the number of simulation repetitions to achieve a desired Monte Carlo standard error. Finally, we give a detailed tutorial on how to apply the ADEMP framework in practice using an example simulation study on the evaluation of methods for the analysis of pre–post measurement experiments.

*Simulation studies are experiments and should be treated as such by authors and editors*.

Hauck and Anderson (1984, p. 215)

## Introduction

Simulation studies are an experimental method for evaluating the properties of statistical methods. They allow researchers to study the statistical properties of methods under complex conditions which would be difficult or impossible to study theoretically, for instance, with formal analyses or mathematical proofs. The idea is to simulate data with known characteristics, analyze these data using the methods under investigation, and then (ideally) compare the results with the known truth. By repeating this procedure under various conditions, the performance and robustness of a method can be assessed and compared to that of other methods. Simulation studies thus represent the “controlled experiment” in the toolbox of methodologists, whereas benchmarking of methods on a real data set would be analogous to a case study. Both are important, but simulation studies in particular allow us to understand when a method works well and when it does not, and ultimately to make recommendations on when to use a particular method in practice. We note that simulation can also be used for other purposes, such as experimental design (e.g., sample size planning or power analysis for complex statistical analyses where no closed-form solutions exist as in Heck & Erdfelder, 2019; Lakens & Caldwell, 2021), statistical inference (e.g., permutation testing or bootstrapping), or numerical integration (e.g., Markov chain Monte Carlo methods for computing posterior distributions in Bayesian statistics), but this use of simulation is typically not called “simulation study” in methodological research and is not the focus of the present paper.

As with any experiment, the quality of evidence from a simulation study depends on how the study is designed, conducted, analysed, and reported. However, unlike many other types of experiments, simulation studies offer much greater flexibility, as it is usually easy and financially inexpensive to change the design of the study and generate new results. This can be seen both as a strength but also as a reason for caution, since there are considerably more *researcher degrees of freedom* than in other types of experiments (Simmons, Nelson, & Simonsohn, 2011). For instance, researchers often have a high degree of flexibility in selecting certain methods and data-generating mechanisms, and in deciding which results are reported. Issues with the conduct and reporting of simulation studies were described almost half a century ago (Hoaglin & Andrews, 1975). However, the attention afforded to researcher degrees of freedom in psychology and other empirical sciences has recently led to more critical reflection on the state of methodological research (Boulesteix, Binder, Abrahamowicz, & Sauerbrei, 2018; Boulesteix, Hoffmann, Charlton, & Seibold, 2020; Boulesteix, Hoffmann, et al., 2020; Friedrich & Friede, 2023; Heinze et al., 2024; Luijken et al., 2023; Pawel, Kook, & Reeve, 2024; Strobl & Leisch, 2022).

Some may argue that simulation studies are often conducted at a more exploratory stage of research and therefore do not require as much rigor and transparency (including measures such as sample size planning, preparation and preregistration of a study protocol, or code and data sharing) as other types of studies. However, many simulation studies are not conducted and reported as exploratory, but rather with the explicit goal of deriving recommendations for the use of methods. It is important to realize that such simulation studies often have a large impact. For example, the simulation study by Hu and Bentler (1999) on cut-off criteria for structural equation models has been cited over 100,000 times, presumably justifying thousands of choices in structural equation modeling. It would be detrimental if the results of such a study were flawed or reported suboptimally. Another example is the simulation study that recommended the “1 variable per 10 events” heuristic as a sample size criterion for logistic regression (Peduzzi, Concato, Kemper, Holford, & Feinstein, 1996). This heuristic has been cited over 8,000 times and was widely adopted as a minimum sample size criterion, but the influential simulation study advocating it was later found to be non-replicable (van Smeden et al., 2016).

In non-methodological research, it has been repeatedly emphasized that research results should be accompanied by measures of statistical uncertainty, such as *p*-values, standard errors, or confidence intervals (Cumming, Fidler, Kalinowski, & Lai, 2012; van der Bles et al., 2019). Clear guidelines are now available in most fields, for example, the APA guidelines require that “*when point estimates [*…*] are provided, always include an associated measure of variability*” (American Psychological Association, 2020, p. 88). It is perhaps surprising, therefore, that methodological researchers rarely report measures of uncertainty associated with the results of simulation studies, even though these researchers tend to be more familiar with statistical reasoning than non-methodological researchers (see the literature reviews by Burton, Altman, Royston, & Holder, 2006; Harwell, Kohli, & Peralta-Torres, 2018; Hauck & Anderson, 1984; Koehler, Brown, & Haneuse, 2009; Morris et al., 2019).

To help navigate the complexities of conducting simulation and benchmarking studies, various guidelines, recommendations and tutorials have been published over the years—both in statistics (e.g., Boulesteix, Groenwold, et al., 2020; Burton et al., 2006; Chipman & Bingham, 2022; Hoaglin & Andrews, 1975; Kelter, 2023; Koehler et al., 2009; Lange, 2022; Morris et al., 2019; White, Pham, Quartagno, & Morris, 2023) and in psychology (e.g., Boomsma, 2013; Carsey & Harden, 2014; Chalmers & Adkins, 2020; Feinberg & Rubright, 2016; Giordano & Waller, 2020; McNeish, Lane, & Curran, 2018; Paxton, Curran, Bollen, Kirby, & Chen, 2001; Psychometric Society, 1979; Skrondal, 2000). A recent tutorial from the statistical literature is provided by Morris et al. (2019), which we recommend as a first read on the state-of-the-art methodology of simulation studies.

A general and accessible introduction to simulation studies that builds on recent guidelines from the statistics literature is currently lacking in psychology. We therefore provide an introduction to the ADEMP (Aims, Data-generating mechanisms, Estimands and other targets, Methods, Performance measure) design and reporting structure of Morris et al. (2019), aimed at researchers in psychology. Based on ADEMP’s structure, we provide a literature review of simulation studies published in the journals *Psychological Methods (PM), Behavior Research Methods (BRM)*, and *Multivariate Behavioral Research (MBR)*, which represent three prominent journals for methodological research in psychology. To help researchers conduct rigorous simulation studies, we provide the ADEMP preregistration (ADEMP-PreReg) template (https://github.com/bsiepe/ADEMP-PreReg) that methodological researchers can use to preregister their simulation studies, clarifying important aspects of their study in advance and helping them to avoid common pitfalls. For simulation studies that are not preregistered, the template can still be used as a blueprint for structured planning and reporting of their study. We also give formulae for the most commonly used performance measures, their Monte Carlo standard errors, and for calculating the number of repetitions to achieve a desired Monte Carlo standard error. Finally, we illustrate ADEMP and the template with an example simulation study on a typical application from psychological research—a comparison of methods for the analysis of pre–post measurements.

## The ADEMP structure

Morris et al. (2019) introduced ADEMP as a structured approach to planning and reporting of simulation studies. Despite its young age, ADEMP has quickly gained traction and is now widely used in (bio)statistics, making it a proof-tested framework. It is important to emphasize that ADEMP is not a legalistic checklist, but a framework for describing the structure of a simulation study. Table 1 provides an overview of ADEMP. We will now summarize the approach in more detail in the context of methodological research in psychology with concrete examples from the field. Readers already familiar with the ADEMP structure may choose to read this section in less detail or to skip it entirely, while readers who want to know more about ADEMP in the context of biostatistics may additionally read Section 3 in Morris et al. (2019). Finally, this section also provides additional recommendations and formulas for planning the number of simulation repetitions based on certain performance measures, and additional recommendations on computational aspects, preregistration, and reporting, that were not discussed in the original article from Morris et al. (2019).

### Aims

The aim of a simulation study refers to the goal of the methodological research project and shapes subsequent choices. Aims are typically related to evaluating the properties of a method (or multiple methods) with respect to a particular statistical task. In psychological simulation studies, common statistical tasks and exemplary aims (taken from the literature review) can include:

- *Estimation*, e.g., assessing the effect of different parametrizations of covariance structures in mixed-effect models when estimating an intervention effect (McNeish & Bauer, 2022).
- *Hypothesis testing*, e.g., comparing different tests of publication bias (Rodgers & Pustejovsky, 2021).
- *Model selection*, e.g., comparing different fit indices for selecting the best structural equation model (Shi, DiStefano, Maydeu-Olivares, & Lee, 2022).
- *Design*, e.g., comparing different methods for determining sample size in mixed-effect modeling (Murayama, Usami, & Sakaki, 2022).
- *Prediction*, e.g., comparing different algorithms for predicting participants’ problem-solving strategies (Moss, Wong, Durriseau, & Bradshaw, 2022).
- *Other aims*, e.g., assessing tools for quantifying complexity (Moulder, Daniel, Teachman, & Boker, 2022), clustering data sets into equivalent parts (Papenberg & Klau, 2021), or comparing implementations of principal component analysis rotations across software packages (Grieder & Steiner, 2021).

These statistical tasks are often closely related, for example, hypothesis testing and model selection may be seen as the same task; the duality of *p*-values and confidence intervals enables both to be used for estimation and hypothesis testing from a frequentist perspective; model selection may be used for the purpose of description, prediction or estimation.

### Data-generating mechanism

The *data-generating mechanism* (often also called *data-generating process*) corresponds to the process of simulating data sets for assessing the performance of the compared methods in accordance with the aims of the simulation study. In general, the data sets can be simulated from a known *parametric model* or by *resampling* an existing real data set. Sometimes part of the simulation uses real data and part simulates data, which is the basis of “plasmode simulation” (Franklin, Schneeweiss, Polinski, & Rassen, 2014; Schreck, Slynko, Saadati, & Benner, 2024; Stolte et al., 2024). For example, data imperfections can be generated in a real-world data set to assess their impact while still preserving part of the structure of the underlying data. (Abrahamowicz et al., 2024).

When simulating from a parametric model, researchers need to specify the data-generating mechanism. For example, the data sets can be generated from a normal distribution with varying values of the underlying true parameters. These can be determined either based on theory and previous research (e.g., depression scores in clinically depressed or healthy populations), estimated from an existing data set, specified according to conventional thresholds (e.g., small, medium, and large effect sizes), or set to arbitrary values to test performance across a wide variety of conditions. A common feature is, however, that the true values of the data-generating mechanism are known by the researcher and can be used to evaluate the performance of the compared methods. Especially when data generation is not based on a real-data model, the choice of data-generating parameters should be explained and justified to enable an understanding of the choice for readers, as well as for researchers wishing to perform similar simulation studies. In some cases this may be “extreme” to see when and how methods break, or not, as the case may be.

If multiple factors are varied, there are different possible ways to combine them: *fully factorial* (all possible combinations), *partially factorial* (considering some combinations but not all possible ones), *one-at-a-time* (varying one factor while holding the other/s constant), or *scattershot* (creating a set of distinct conditions). The fully factorial approach is typically preferred because it allows us to disentangle the individual effects of the factors and their interactions, but it may not always be feasible computationally or because some combinations of factors make no sense. For example, in a simulation study involving missing data, we may wish to vary the proportion of missing data and the missing data mechanism. When the proportion is zero, the mechanism is not applicable. Complex simulation designs can also make the reporting and interpretation of results more difficult. To reduce the complexity of the design, a partially factorial design may then be chosen (Morris et al., 2019, see, for example, Skrondal (2000) for recommendations on “fractional factorial designs”).

**Figure 1:**
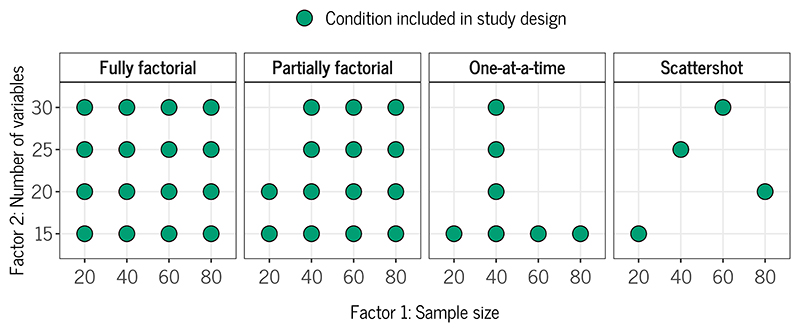
Example of Different Ways to Combine Factors in the Design of a Simulation Study.

Figure 1 gives an example of how two factors, sample size and the number of variables, could be combined for a simulation study comparing different regression methods: The fully factorial approach would include all possible combinations (left panel). However, this may not be possible because, for example, the regression methods under study may not be able to handle situations where the number of variables is greater than the sample size. In this case, these conditions may be excluded and a partially factorial design adopted (middle left panel). With the one-at-a-time approach, one may fix the sample size to a value of 40 and then vary the number of variables across all levels, and vice versa, fix the number of variables to 15 and vary the sample size across all levels (middle right panel). Finally, with the scattershot approach, one may create distinct conditions of sample size and number of variables, for example, inspired by actual data sets that feature these combinations (right panel). Depending on the setup of this approach, higher-order interaction effects between simulation factors may not be identifiable.

When resampling an existing data set, researchers rely on a (usually large) existing data set to sample smaller data sets for the simulation. Alternatively, one may sample equally large data sets with replacement from the existing data set. The data-generating mechanism is thus implicitly determined by the data set while researchers only need to specify the resampling mechanism.

### Estimands and other targets

Estimands and other targets jointly refer to the practical aims of the compared methods, Table 2 provides an overview of common targets of simulation studies. For example, if a simulation study aims to compare different methods for estimating the effect of an intervention *versus* the absence of that intervention, the estimand of interest is a contrast of these groups rather than, say, a group mean. An estimand is a target quantity of a statistical analysis (see ICH, 2019; Keene, Lynggaard, Englert, Lanius, & Wright, 2023; Lundberg, Johnson, & Stewart, 2021, for accessible introductions to estimands). In simulation studies, an estimand is typically, but not always, a parameter of the underlying data-generating model. When it is not, care is needed to define and compute the true or ideal value of an estimand. If the simulation study aims to compare different methods for hypothesis testing rather than estimation, the true hypothesis is the target of interest.^1^ Again, care is needed to distinguish between different ways of translating substantive hypotheses into statistical hypotheses (e.g., whether a null hypothesis of no effect is specified as a point null hypothesis or an average null hypothesis in random-effects meta-analysis). Similarly, the targets appropriate for the statistical tasks of other simulation studies include the true model (when the statistical task is model selection), the design characteristics (when the statistical task is design), or new data (when the statistical task is prediction).

### Methods

The term “methods” corresponds to the different procedures evaluated in the simulation study for estimating parameters, testing hypotheses, predicting new data, etc. There is some ambiguity about what exactly constitutes a method in simulation studies. Often, different methods correspond to different statistical procedures, e.g., different tests for assessing publication bias such as the Egger regression or selection-model tests. Methods can also encompass different specifications or settings of a single statistical procedure, e.g., different parameterizations of covariance structures in mixed-effect models, different metrics of a statistical procedure, e.g., fit indices, or different software implementations of the same procedure, or the performance of different variance estimators for a given point estimator, e.g. model-based, robust, and bootstrap based standard errors. Less often, a simulation study evaluates only a single method, for instance, to verify that the method can recover the targeted estimands in the first place or to study robustness when its assumptions are violated. Of course, it may also be the case that no competing method is available.

If only a single method is evaluated and there is no clear benchmark for what constitutes “good” performance (e.g., the successful control of the Type I error rate at 5% in the case of a statistical test), the lack of a comparator method can introduce ambiguity into a simulation study. If multiple methods are compared, caution is required along various steps of planning and conducting a simulation study to ensure that comparisons are both neutral and meaningful. As in research on new drugs or treatments, methodological researchers have warned against over-optimism in the evaluation of new statistical methods for some time (e.g., Boulesteix, 2015). Such over-optimism can occur, among other reasons, when researchers are not neutral with respect to the evaluation of a method but rather choose data-generating mechanisms that favor a certain method (Nießl, Hoffmann, Ullmann, & Boulesteix, 2023). Comparisons aimed at identifying data characteristics that determine the performance of the investigated methods (and their contextualized relative advantages) are often more informative than searching for specific conditions under which a method appears to be the “best” (Strobl & Leisch, 2022).

Comparative simulation studies can benefit from approaches that decrease over-optimism and allegiance bias used in other scientific fields such as experimental psychology or clinical trials. These include blinding the data analysts to the method (Pawel et al., 2024) or using separate research teams for data simulation and analysis (Kreutz et al., 2020). Further, “adversarial collaboration”, the collaboration of researchers with different theoretical or methodological views (Cowan et al., 2020; for an example see Binder, Sauerbrei, & Royston, 2012), could be introduced to simulation studies to achieve useful comparisons between different methods. Researchers can also build on previous research by combining the conditions and methods of previous simulation studies into a single, large simulation study, extending previous simulation designs when necessary, to assess the robustness of their results to different experimental settings that have already been investigated in isolation by others (see Bartoš, Maier, Wagenmakers, Doucouliagos, & Stanley, 2023; Hong & Reed, 2021, for an example).

### Performance measures

Performance measures are the summary statistics used for quantifying how well methods can achieve their task for a given data-generating mechanism. For instance, a performance measure may quantify how well a method can estimate an estimand. As such, the estimated performance corresponds to the “inferences” of a simulation study that allow researchers to draw conclusions about the methods. The selection of appropriate performance measures depends on the aims of the simulation study, but also the estimands and other targets. For example, bias, (root) mean square error, and confidence interval coverage can be used to evaluate methods for estimating intervention effects, while power and Type I error rate might be used to evaluate methods for testing hypotheses about publication bias. Table 2 shows typical performance measures for different simulation study aims.

The same statistical method may be applied for different statistical tasks and in different contexts, such as estimation and prediction, for which different performance measures can be used. Typically, multiple performance measures for a method should be interpreted together (Morris et al., 2019). For example, one may only consider comparing the statistical power of different hypothesis testing methods if these methods have appropriate Type I error rates (e.g., are below 5%). When evaluating estimation performance, it is often desirable to interpret the bias and variance of an estimator together, as there is typically a trade-off between the two. In general, providing a rationale for the choice of performance measure as well as defining it clearly (ideally, with a formula-based representation) avoids ambiguity. This is especially important when less familiar performance measures are used, and when performance is estimated conditional on some sample statistic (e.g., bias of a study given that it converged in a given simulated data set).

Performance measures used in simulation studies are typically aggregated across all simulation repetitions. For example, the bias is estimated as the mean deviation between parameter estimates and the true parameter across all repetitions. It can often be informative, especially when building and reviewing a simulation study, to also look at other quantities than the mean, for example, the median or other quantiles, or to visualize the distribution, for example, with violin or box plots of parameter estimates, *p*-values, or Bayes factors. This strategy may be useful for two reasons. First, it can help uncover errors in the simulation design if the distribution of performance estimates violates expectations from theoretical work or other simulation studies. Second, properties such as large variability, skewness, or multimodality of the distribution of performance estimates can provide important information about the performance of a statistical method that is not captured by the mean.

An important aspect that affects the estimation and interpretation of performance measures is the convergence of methods. By “convergence”, we mean broadly that a method successfully produces the outcomes of interest (e.g., an estimate, a prediction, a *p*-value, a sample size, etc.) required for estimation of performance. Although convergence may not always be the main interest of a simulation study, it should be reported whether or not non-convergence occurred, and if so, under what conditions and for which methods. Non-convergent repetitions are the “missing values” of a simulation study, and they impact the interpretation of other measures, since these can only be estimated when a method converged. This is complicated because one method may converge more often than others, and so the comparison conditional on convergence is delicate. The way non-convergent repetitions are handled can have a major impact on results and conclusions, especially if repetitions are not missing at random. For example, if a method fails to converge under the most challenging conditions (e.g., small sample sizes), excluding such repetitions only for the problematic method while keeping the results for all other methods that did converge may bias performance measure estimates in favor of the excluded method. In such a case, researchers should explicitly report and investigate patterns of non-convergence (Chalmers & Adkins, 2020; Giordano & Waller, 2020).

Table 3 provides definitions of the performance measures that were most commonly used in our literature review of simulation studies in psychology (which will be presented in the next section). We refer to Table 6 in Morris et al. (2019) for a definition and Monte Carlo standard errors (MCSEs) of various other performance measures, such as bias-eliminated coverage or average model standard error, that are less frequently used in psychology. Performance measures estimated from simulated data are subject to sampling variability, similarly to any other quantity estimated from a finite set of data (Koehler et al., 2009). Much like the sample size in other empirical studies, the number of simulation repetitions *n*_sim_ determines the precision of these estimates. Table 3 therefore provides formulae for approximate MCSEs associated with the estimated performance measures. All MCSEs are based on the assumptions of independent simulations and approximate normality of the estimated performance measures. More accurate jackknife-based MCSEs are available through various R packages such as rsimsum (Gasparini, 2018) and simhelpers (Joshi & Pustejovsky, 2022). The SimDesign R package (Chalmers & Adkins, 2020) can compute confidence intervals for performance measures *via* bootstrapping.

MCSEs (or other measures of uncertainty, including visual representations) should be provided alongside the estimates of performance to indicate the associated uncertainty. Failing to calculate and report Monte Carlo uncertainty can lead to erroneous interpretations of results and unsupported claims about the performance of different methods (see, e.g., the illustration by Koehler et al., 2009). In situations where MCSEs are tiny relative to the estimated performance and may distract, one could, for example, provide the maximum MCSE across all conditions to give the reader reassurance about the worst case.

When planning a simulation study, researchers should choose a number of simulation repetitions that ensures a desired precision for estimating the chosen performance measures. The last column of Table 3 gives simple formulae for this purpose. Many of these depend on quantities that are not known but have to be estimated from the simulated data. For example, the MCSE of the estimated coverage depends on the coverage itself. In this case, one can either assume a certain value for which the desired MCSE should be achieved (e.g., 95%), take a “worst-case value” in the sense that the MCSE is maximal for a given number of repetitions (this would be 50% for coverage), or estimate it from a small pilot study (e.g., taking the estimated coverage closest to 50% across all conditions and methods obtained in the pilot study). The latter approach may be especially advisable for performance measures where there is no conventional benchmark, such as 95% is for coverage.

In practice, it can be challenging to define what it means for an MCSE to be “sufficiently small”. A. S. Cohen, Kane, and Kim (2001) provide some guidelines on how to decide on the desired precision based on the size of the effects under study. Essentially, the number of repetitions must be chosen large enough such that the MCSE is sufficiently small compared to the relevant effect of interest (e.g., if a change in coverage of 1% is a relevant effect, the MCSE for the estimated coverage should be less than that). However, what exactly constitutes a relevant effect must be decided by researchers on a case-by-case basis, as to our knowledge there are no standards. This parallels the challenges in traditional sample size calculations, where researchers must also decide on a minimum effect size of interest.

Finally, during the design phase of a simulation study with clear expectations about the performance of different methods, researchers may also wish to specify in advance what constitutes a “relevant difference” in performance, or what constitutes “acceptable” and “unacceptable” levels of performance, to avoid post-hoc interpretation of performance. Such studies may be seen as “confirmatory” methodological research (Herrmann et al., 2024). For example, it could be stated that a Type I error rate greater than 5% defines unacceptable performance, or that a method X is considered to perform better than a method Y in a given simulation condition if the estimated performance of method X minus its MCSE is greater than the estimated performance of method Y plus its MCSE. Again, this is similar to traditional sample size calculations where researchers need to decide on a minimum effect size of interest they want to detect (Anvari & Lakens, 2021). While this can be difficult in practice, it forces researchers to think thoroughly about the problem at hand, so investing this time comes with the benefit of higher clarity of expectations and interpretation.

### Reporting

As with any experiment, transparent reporting of study design, execution, and results is essential to put the outcomes from a simulation study into context. The ADEMP structure is a useful template for researchers to follow when reporting the design and results of their simulation study. Furthermore, the results should be reported in a way that clearly answers the main research questions and acknowledges the uncertainty associated with the estimated performance. It is often difficult to find a balance between streamlining the results of simulation studies for the reader and exhaustively reporting all conditions in detail. However, it is important that researchers avoid selectively reporting only certain conditions that favor their preferred method or are in line with their expectations, as this can lead to overoptimism (Pawel et al., 2024).

Figures are often helpful for interpreting large quantities of results and identifying general trends. However, for most plot types, there is a limit to how many factors can be communicated visually (see section 7.2 in Morris et al., 2019, for some recommendations, see also Rücker & Schwarzer, 2014). On the other hand, presenting results only with figures can hinder the accurate interpretation of results and also make it more difficult for researchers replicating the simulation study to verify whether they have been successful (Luijken et al., 2023). Figures should therefore ideally be combined with quantitative summaries of results, such as tables or graphical tables containing both numerical and graphic elements.^2^ For complex simulation designs with a large number of conditions, the communication of results can be improved using interactive tools such as R Shiny applications (Chang et al., 2023, see e.g., Carter, Schönbrodt, Gervais, & Hilgard, 2019; Gasparini, Morris, & Crowther, 2021).

### Computational aspects

The computational implementation of simulation studies can often be complex. Conclusions critically depend on the soundness of the underlying code for data-generation, model fitting, and computations of performance measures, and even small mistakes can have a big impact (Schwab & Held, 2021). Morris et al., 2019 and White et al., 2023 give detailed advice on how to code a simulation study, here we want to focus more on the aspects of reproducibility and code sharing. Code that is not openly available online prevents an assessment of the computational reproducibility of simulation studies. It is also an obstacle for reviewers and readers who want to understand, inspect, or replicate the implementation of a simulation study, or for researchers who seek to build on the previous literature. It is therefore recommended for researchers to make their code openly available, share all relevant information about their computational environment, and strive to use a robust computational workflow to ensure the reproducibility and replicability of their results (Chalmers & Adkins, 2020; Giordano & Waller, 2020; Luijken et al., 2023; Pawel et al., 2024).

Platforms such as the Open Science Framework and Zenodo can be used to persistently store and share data and code, independently of specific journals and according to the FAIR principles (Wilkinson et al., 2016). The computational reproducibility of simulation studies can be further enhanced by sharing complete or intermediate results of simulation studies, such as the simulated data or parameter estimates of computed models. This enables independent reproduction and evaluation of the results by other researchers without the full computational effort that large simulation studies require.

Information on the computational environment and operating system is relevant to reproduce simulation studies. Different software packages or package versions can lead to different results, even when the apparently same method is used (Hodges et al., 2023). Operating systems can differ in a variety of aspects that may subtly influence the results of analyses (Glatard et al., 2015). There are several helpful tools that facilitate sharing information on the computational environment and operating system. For example, when using R, the output of the sessionInfo() command includes information about the operating system, R package versions, and auxiliary dependencies (e.g., the installed linear algebra programs such as BLAS/LAPACK). Furthermore, Peikert and Brandmaier (2021) and Epskamp (2019) provide accessible tips for reproducible workflows in R, which can serve as a starting guide for other statistical software as well. For instance, in advanced workflows, a snapshot of the current version of all software required to reproduce the analysis is stored (e.g., via Docker or the R package renv, Ushey & Wickham, 2023).

An important computational aspect of simulation studies is the use of pseudo-random numbers. It is important to initialize the random number generator with a seed and to store this seed so that the same sequence of pseudo-random numbers can be reproduced in the future (assuming other dependencies, such as operating system and software versions, remain the same). The primary purpose of the seed is to ensure computational reproducibility and to facilitate debugging. At the same time, the seed should not matter for simulation studies with a sufficient number of repetitions, because the seed should have a negligible effect on the results (estimated performance measures, patterns, and conclusions). Things become more complicated when multiple cores, clusters, or computers are used for running the simulation study since the seed has to be set for each parallelized instance to ensure reproducibility. One solution is to use “streams” rather than seeds, which fixes the random number generator to the actual starting position in the deterministic sequence of generated numbers (Morris et al., 2019). Streams are available in Stata, SAS, and R^3^. However, when using streams, one needs to know how many pseudo-random numbers are required per instance, so that the streams can be set to avoid overlap. This can be challenging, especially when the methods evaluated in the simulation study also use pseudo-random numbers (e.g., Markov Chain Monte Carlo sampling or bootstrap methods).

## Literature review

**Figure 2:**
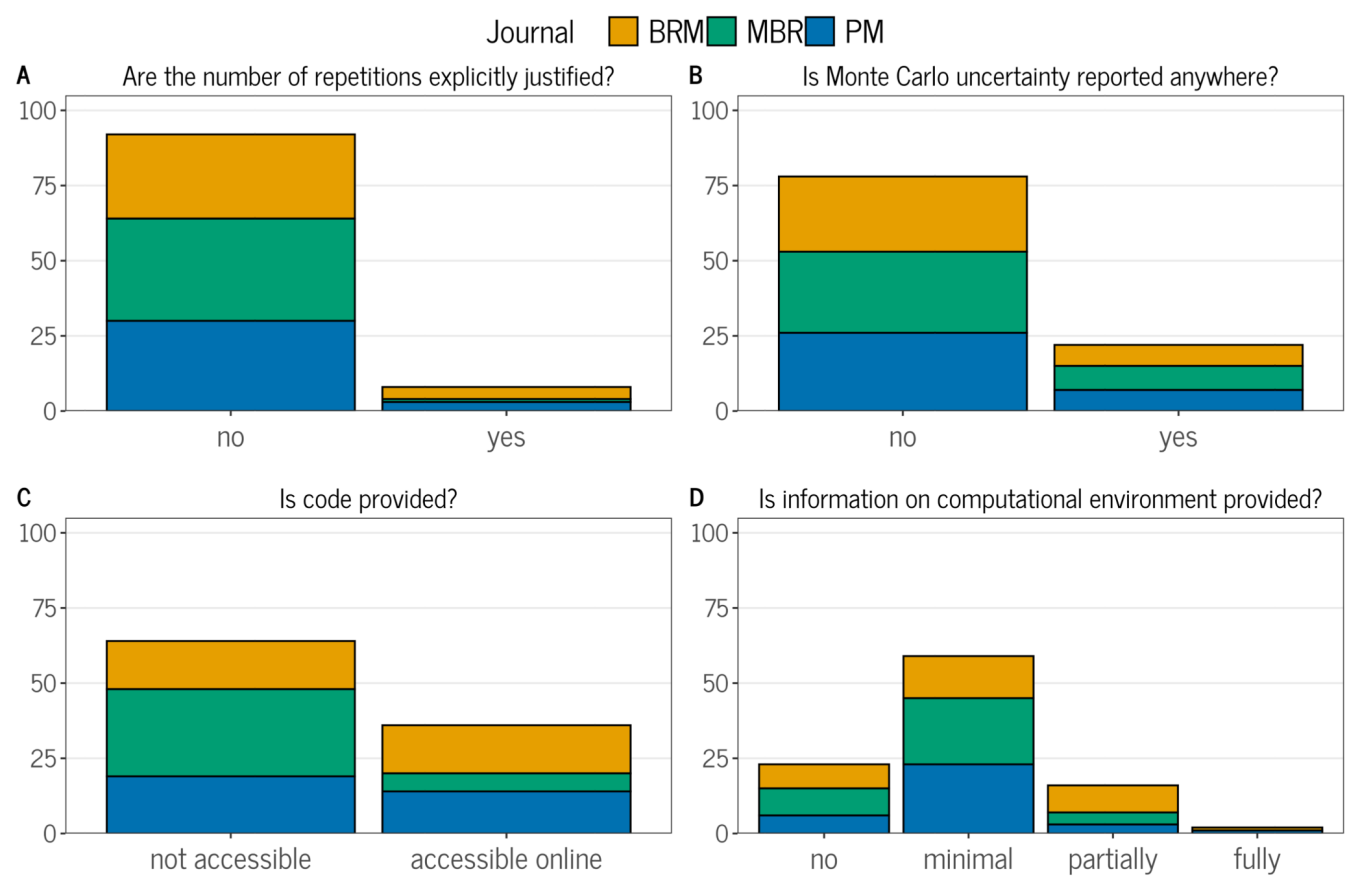
Common Issues of Simulation Studies in Psychology as Identified in the Literature Review. *Note*. 100 articles were reviewed that included simulation studies and were published in *Psychological Methods, Behavior Research Methods*, and *Multivariate Behavioral Research* in 2021 and 2022.

**Figure 3:**
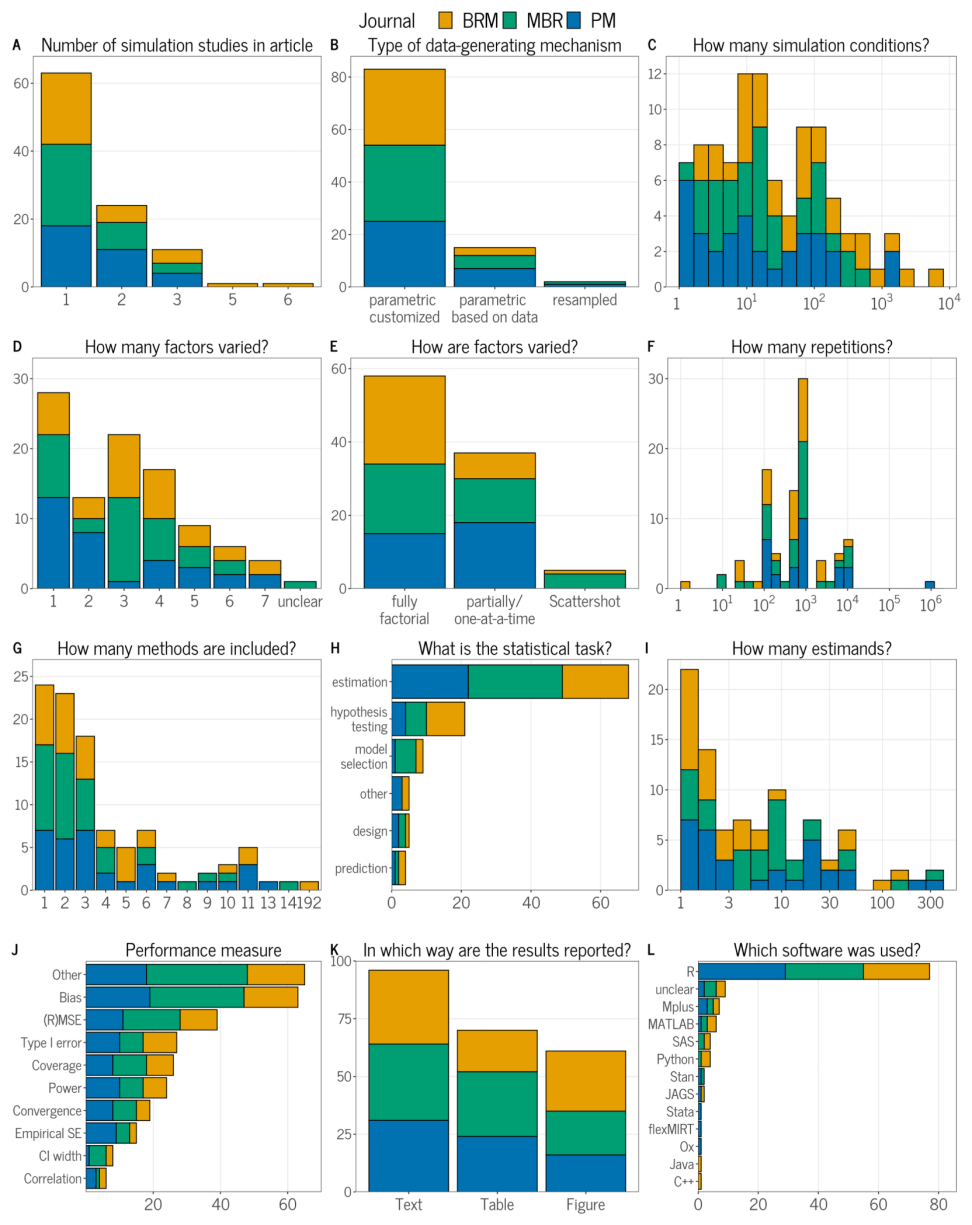
Descriptive Results from Literature Review of Simulation Studies in Psychology. *Note*. 100 articles were reviewed that included simulation studies and were published in *Psychological Methods, Behavioral Research Methods*, and *Multivariate Behavioral Research* in 2021 and 2022. In Panel J, absolute and relative bias are combined in the bias category. In Panel E, partially factorial and one-at-a-time are combined. Within-panel totals are greater than 100 in panels H, J, K, L due to the possibility of more than one category.

In this section, we use the ADEMP structure to assess the current state of simulation studies in psychology. For each ADEMP component, we summarize the findings, highlight their relevance, and suggest improvements for future simulation studies. We compare some of our results with the results of Morris et al. (2019) who reviewed 100 simulation studies published in *Statistics in Medicine*. Visual summaries of the review are provided in Figure 2 and Figure 3. Table 4 summarizes the most common pitfalls we encountered during the review. The preregistration, data, and code to reproduce the results are available at the Open Science Framework (https://osf.io/dfgvu/).

We extracted 321 articles until we reached 100 articles containing at least one simulation study. We extracted articles by going through the 2022 issues of the journals in chronological order. After assessing the number of articles containing a simulation study from each journal, we then continued chronologically in the 2021 issues, aiming for a roughly equal split of simulation studies from the three journals.^4^ The proportion of articles containing a simulation study (31.2%) was considerably lower than the 75.4% proportion reported by Morris et al. (2019) for the 100 simulation studies published in *Statistics in Medicine*. The lower proportion of simulation studies in our review is mainly due to articles in *BRM*, which generally published the most articles, but only 15.6% of them contained a simulation study. We extracted roughly equal numbers of articles containing a simulation study from the three journals, with 32 from *BRM* and 35 each from *PM* and *MBR*. Of these articles, 63 contained only a single simulation study, while the rest contained up to 6 simulation studies (see Panel A of Figure 3).

Three authors (BS, FB, SP) each reviewed around one-third of all simulation studies and assessed the overall confidence in their rating of each study as “low”, “medium”, or “high”. To assess inter-rater agreement, each rater also reviewed six studies that were assigned to the other raters and which had a “low” or “medium” confidence rating, thereby representing the most challenging simulation studies that were reviewed. Nevertheless, an agreement larger than 75% was achieved for the majority of questions (Median = 83.3%). The lowest agreement was with respect to whether the estimands were stated and the number of estimands (above 30%). All studies where we disagreed about the number of estimands were studies about latent variable models, where it was often unclear which parameters were of interest and how their number varied across conditions, with many studies even showing varying numbers of parameters per condition. The results of the agreement analysis are shown in Figure 5 in the Appendix.

### Aims

In 94% of the reviewed articles, the aims of the study were defined in some form. We did not quantify how specific or vague the aims were defined, although they were often defined rather vaguely (“We conducted a simulation study to evaluate the performance of method X”). By far most studies had estimation as one of their statistical tasks (68%), followed by hypothesis testing (21%) and model selection (9%; Panel H in Figure 3). This resembles the results of Morris et al., who also found these three tasks to be the most prominent ones with similar frequencies.

### Data-generating mechanism

In our review, the clear majority of simulation studies (83%) generated data based on parametric models with parameters specified by researchers (‘parametric customized’), while 15% were directly based on parameter estimates from real data (Panel B of Figure 3). The remaining 2% used resampling techniques. In almost all of the studies (95%), the data-generating parameters were provided, which mirrors the results from Morris et al. (91% studies). Nevertheless our view is that many of the reviewed papers could have benefited from describing the data-generating mechanism in a more structured way to facilitate easy comprehension and replication.

Researchers used between 1 and 6,000 simulation conditions (Median = 16; Panel C in Figure 3). In these, they varied between 1 and 7 factors, with 1 and 3 being the most common choices (Panel D in Figure 3). Of all designs, 58% were fully factorial, meaning that all possible combinations of factor levels were investigated. Moreover, 37% of the studies were either partially factorial or varied factors one-at-a-time (including studies with a single design factor) and 5% used distinct scenarios in a scattershot design (Panel E in Figure 3). As in experimental psychology, a fully factorial design enables the study of the main and interaction effects of the varied factors. In our review, some studies made use of this fact by using analysis of variance to assess the effects of simulation factors (see also Chipman & Bingham, 2022).

The number of repetitions per simulation condition ranged between 1 and 1,000,000 (Panel F in Figure 3). The median number was 900, whereas the most frequently selected options were 1,000 repetitions followed by 500 repetitions, similar to the results from Morris et al.. However, in 17% of studies, at least some of the performance results were aggregated across multiple parameters (such as the average bias across factor loadings), leading to higher precision. Only 8% of the studies provided a justification for the specific number of repetitions used, while only 3% of these actually performed a calculation of the required number of repetitions (Panel A in Figure 2). This is very similar to the results from Morris et al., who also found only 4% of studies presenting a justification for their choice of the number of repetitions. This lack of justification is, unfortunately, consistent with the findings from similar surveys of the methodological literature (Harwell et al., 2018; Hauck & Anderson, 1984; Hoaglin & Andrews, 1975; Koehler et al., 2009). Of course, this does not rule out the possibility that the study authors chose their number of repetitions in some informed way (e.g., by visually assessing whether Monte Carlo uncertainty was sufficiently small) without explicitly reporting their rationale.

### Estimands and other targets

In 20% of the studies, the estimands or targets of the simulation were either not reported or unclear to us. Of those that were clear, most studies focused only on a single estimand, while the median number of estimands was 4. In at least 17% of the studies, estimated performance measures related to different estimands were later aggregated to calculate average performance, while this was unclear in 4% of studies. We noticed that especially when evaluating models with many parameters, such as latent variable models or certain time series models, it can easily become unclear which parameters are of interest. Clear definition and reporting of estimands and (potentially aggregated) performance measures is particularly important in these situations.

### Methods

While the number of methods evaluated in the simulation studies ranged from 1 to 192, more than half (65%) evaluated 3 or fewer methods, and 24% evaluated only a single method (Panel G in Figure 3).

### Performance measures

Reflecting the popularity of estimation as a statistical task, bias (used in 63% of studies) and (root) mean square error ([R]MSE, in 39% of studies) were the most common performance measures in our review (Panel J in Figure 3). Convergence was reported only in 19% of the studies. This is problematic because substantial non-convergence can greatly affect the conclusions of simulation studies (van Smeden et al., 2016). In 10% of the studies, performance measures were unclear, for example, how a certain performance measure was defined mathematically. Many studies also included other performance measures not explicitly listed here. For example, the correlation between true and estimated parameters was sometimes used as a measure of performance. While there may be cases where this metric provides valuable information, interpreting it without considering the bias and variance of the estimates gives only a very limited insight into the performance of a statistical method. A positive example of clear reporting of both model specifications and performance measures is H. Liu, Yuan, and Wen (2022), who provided formulae for both their models and the performance measures used.

### Presentation of results

Simulation results were most commonly reported in the text of an article and accompanied by tables and figures (Panel K in Figure 3). The vast majority of studies (77%) did not report the uncertainty of performance measures (Panel B in Figure 2), despite our liberal approach of including visualizations such as box plots as indicative of Monte Carlo uncertainty. The proportion is comparable to the stricter approach of Morris et al. (2019) who counted 93% of their studies not reporting Monte Carlo standard errors. To cite two positive examples from our review, J. Liu and Perera (2022) ran a pilot simulation study to obtain the empirical standard errors for parameter estimates, which they then used to calculate the needed number of repetitions to keep the MCSE below a desired level. Rodgers and Pustejovsky (2021) provided the upper bound of the MCSE of their performance measures to indicate their precision.

### Computational aspects

R was the most commonly used statistical software to conduct simulation studies and was used in 77% of the studies (Panel L in Figure 3). Notably, the software used was unclear or not mentioned in 9% of the studies. In Morris et al. (2019), 38% of the studies did not mention the software used for their simulation study. In around half of the studies we reviewed, authors also indicated that they used some form of user-written commands, such as custom model code, or packages for their simulations. To fully understand these simulations, it would be crucial to share code alongside the manuscript. However, code was not available for almost two-thirds of the simulation studies (64%; Panel C in 2). This also includes cases in which code was supposed to be provided, but the repository was not available, and cases in which code was supposedly available “upon (reasonable) request”. In multiple cases, authors supposedly provided code on the journal website or on a university homepage, but the code was not available at the designated location. Our results are similar to the findings of Kucharský, Houtkoop, and Visser (2020), who analyzed articles in three methodological journals (including *Psychological Methods* and *Behavior Research Methods*) and found that 56% of studies that contained coded analyses did not share their code. Of the 36% of studies in our review in which code was provided, 21% also provided a seed in their code. We did not check if this seed and the supplied code would be sufficient to reproduce the reported results.

Beyond the code and software used, we reviewed whether articles contained information on the computational environment and operating system used. We coded information on the computational environment as “fully” when packages with versions and auxiliary dependencies were provided, for example in a “sessionInfo” output from R or via a Docker container. We rated the information as “partially” or “minimal” when the main packages used were reported with or without versions, respectively. Full information on the computational environment was only reported in 2% of the studies, while 24% did not report on their computational environment at all (Panel D in Figure 2). Even more studies (93%) did not provide any information whatsoever on their operating system. Full information (naming the operating system and its version) was provided in 4% of the studies, while 3% at least provided the operating system without stating its version. Papenberg and Klau (2021) are a positive example that included full information on their computational environment and operating system which they used, as well as code and data to reproduce the simulations.

## The ADEMP-PreReg template

Our literature review highlights varying standards of reporting of simulation studies in psychology. To promote more structured and detailed reporting and to simplify preregistration of simulation studies, we developed the ADEMP-PreReg template. This template closely follows the outlined ADEMP structure and provides a list of questions, their explanation, and example answers. This prompts researchers to describe all relevant parts of their simulation studies a priori. The template can additionally be used as a blueprint for reporting or as guidance when reviewing simulation studies. As such, the ADEMP-PreReg template is not only suited for experienced researchers who want to plan, preregister, or report their simulation studies but also for (under)graduate students embarking on a first exploration of simulation studies. Once a simulation study is conducted, the ADEMP-PreReg template can be transformed into the method section of the simulation study with minimal effort. The template is available at GitHub (https://github.com/bsiepe/ADEMP-PreReg) in different versions (LATEX, Microsoft Word, Google Docs, Overleaf) and can be uploaded and timestamped at OSF, AsPredicted, or Zenodo among others. The ADEMP-PreReg template is intended as a “living document” and we welcome feedback and suggestions via opening an issue or a pull request on the GitHub repository.^5^

It is worth emphasizing that preregistration of simulation studies has many parallels, but also several differences to the preregistration of other studies. Traditionally, preregistration is often used to distinguish between exploratory and confirmatory research. This distinction is more blurry for simulation studies, as it is often not clear when “data collection” starts and which part of the research process can be considered confirmatory. This parallels the challenges with preregistrations of secondary analyses on observational data (den Akker et al., 2021). At the same time, preregistration in the traditional sense may be more appropriate in later stages of methodological research (Heinze et al., 2024) when researchers attempt to neutrally compare already well-established methods. Beyond that, preregistration of simulation studies can serve many other purposes. It can help to structure the planning of a study, guard against cognitive biases in the interpretation of results, promote a minimum degree of neutrality and transparency, and save work in the preparation of manuscripts. As in other areas of empirical science, preregistration may also help to legitimize and publish informative “null” results in methodological research. Even if researchers do not want to preregister their simulation, the preregistration template can serve as a blueprint for planning and reporting. Finally, for readers, particularly journal editors and peer reviewers, the ADEMP protocol can serve as a guiding document (similar to McNeish et al., 2018). By considering whether the questions in the protocol can be answered after reading a manuscript, reviewers can assess whether all necessary information is included and communicated in an accessible manner.

### Example simulation study on methods for the analysis of pre–post measurements

To illustrate the application of the preregistration template, we conduct a simulation study to evaluate different methods for analyzing data from pre–post measurement experiments. The filled-out ADEMP-PreReg template is available at https://osf.io/dfgvu/. We made a minor modification of the study design from the preregistration, increasing the number of simulation repetitions to achieve a lower MCSE than was originally planned since our preregistration did not guarantee a sufficiently small MCSE for the worst-case power. Importantly, the sole purpose of this simulation study is to illustrate both the template and the transparent reporting of results using easy-to-understand simulation conditions, not to contribute new knowledge to the literature. See Clifton and Clifton (2019); Lüdtke and Robitzsch (2023); Senn (2006); Van Breukelen (2013); Vickers (2001) for a comprehensive treatment of the topic.

#### Aims

The aim of the simulation study is to evaluate the hypothesis testing and estimation characteristics of different methods for estimating the treatment effect in pre–post measurement experiments. We compare three different methods (ANCOVA, change score analysis, and post score analysis) in terms of power and Type I error rate related to the hypothesis test of no effect, and bias related to the treatment effect estimate. We vary the true treatment effect and the correlation of pre- and post-measurements.

#### Data-generating mechanism

In each simulation repetition, we generate *n* = 50 pre–post measurements in the control group (*g* = control) and *n* = 50 pre–post measurements in the experimental group (*g* = exp) from a bivariate normal distribution

(1) $$\left[\begin{array}{c} Y_{1}\\ Y_{2} \end{array}\right]∼\text{N}\left(\left[\begin{array}{c} 0\\ \mu_{g,2} \end{array}\right],\left[\begin{array}{c} 1\;\rho \\ \rho \;1 \end{array}\right]\right),$$

where the first argument of the normal distribution in (1) is the mean vector and the second argument the covariance matrix. The numerical subscript 1 indicates measurement time “pre” and 2 indicates “post”. The parameter *µ*_*g*,2_ denotes the post-treatment mean. It is fixed to zero in the control group (*µ*_control,2_ = 0), whereas it is varied across simulation conditions in the experimental group. The parameter *ρ* denotes the pre–post correlation and is also varied across simulation conditions.

We use the following values for the manipulated parameters of the data-generating mechanism:

- *µ*_exp,2_ ∈ {0, 0.2, 0.5}
- *ρ* ∈ {0, 0.5, 0.7}

We vary the conditions in a fully factorial manner which results in 3 (post-treatment mean in experimental group) *×* 3 (pre–post measurement correlation) = 9 simulation conditions. We select the specific values as they correspond to the conventions for no, small, and medium standardized mean difference effect sizes in psychology (J. Cohen, 2013). The pre–post measurement correlations correspond to no, one quarter, and approximately one half of shared variance that, based on our experience, are both realistic and also allow us to observe differences between the examined methods.

#### Estimands and other targets

Our primary target is the null hypothesis of no difference between the outcomes of the control and treatment groups. Our secondary estimand is the treatment effect size defined as the expected difference between the control and the experimental group measurements at time-point two

$$\text{E}\left(Y_{2}|g=\text{exp}\right)-\text{E}\left(Y_{2}|g=\text{control}\right),$$

for which the true value is given by the parameter *µ*_exp,2_ for the considered data-generating mechanisms.

#### Methods

We compare the following methods:

1. **ANCOVA** (ANalysis of COVAriance): A regression of the post-treatment measurement using the pre-treatment measurement and the treatment indicator as covariates, which is specified in **R** as lm(post ∼ pre + treatment)
2. **Change score analysis**: A regression of the difference between post-treatment and pre-treatment measurement using the treatment indicator as covariate, which is specified in **R** as lm(post ∼ offset(pre) + treatment)^6^
3. **Post score analysis**: A regression of the post-treatment measurement using the treatment indicator as covariate, which is specified in **R** as lm(post ∼ treatment)

Both change score and post score analysis can be seen as special cases of ANCOVA. Change score analysis fixes the pre coefficient to 1 (using the offset() function), and post score analysis omits the pre variable from the model (effectively fixing its coefficient to 0).

#### Performance measures

Our primary performance measures are the Type I error rate (in conditions where the true effect is zero) and the power (in conditions where the true effect is non-zero) to reject the null hypothesis of no difference between the control and treatment condition. The null hypothesis is rejected if the two-sided *t*-test *p*-value for the null hypothesis of no effect is less than or equal to the conventional threshold of 0.05. The rejection rate (the Type I error rate or the power, depending on the data generating mechanism) is estimated by

$$\hat{\text{RRate}}=\frac{\sum_{i=1}^{n_{\text{sim }}}𝟙\left(p_{i}\leq 0.05\right)}{n_{\text{sim}}}$$

where 𝟙(*p*_*i*_ ≤ 0.05) is the indicator of whether the *p*-value in simulation *i* is equal to or less than 0.05. We use the following formula to compute the MCSE of the estimated rejection rate

$${\text{MCSE}}_{\hat{\text{RRate}}}=\sqrt{\frac{\hat{\text{RRate}}(1-\hat{\text{RRate}})}{n_{\text{sim}}}}.$$

Our secondary performance measure is the bias of the treatment effect estimate. It is estimated by

$$\hat{\text{Bias}}=\frac{\sum_{i=1}^{n_{\text{sim }}}{\hat{\theta }}_{i}}{n_{\text{sim}}}-\theta$$

where *θ* is the true treatment effect and ${\hat{\theta }}_{i}$ is the effect estimate from simulation *i*. We compute the MCSE of the estimated bias with

$${\text{MCSE}}_{\hat{\text{Bias}}}=\frac{S_{\hat{\theta }}}{\sqrt{n_{\text{sim}}}}$$

where $S_{\hat{\theta }}=\sqrt{\frac{1}{n_{\text{sim}}-1}\sum_{i=1}^{n_{\text{sim }}}{\left\{{\hat{\theta }}_{i}-\left(\frac{1}{n_{\text{sim}}}\sum_{i=1}^{n_{\text{sim }}}{\hat{\theta }}_{i}\right)\right\}}^{2}}$ is the sample standard deviation of the effect estimates.

Based on these performance measures, we perform 10,000 repetitions per condition. This number is determined by using the formulae from Table 3 in Siepe et al. (2023) aiming for 0.005 MCSE of Type I error rate and power under the worst case performance (50% rejection rate: 0.50 *×* (1 − 0.50) / 0.005^2^ = 10,000), which we deem to be sufficiently accurate for estimating power and Type I error rate for all practical purposes. Our simulation protocol also illustrates how to determine the number of repetitions for bias based on a small pilot simulation study to estimate the unknown effect estimate variance.

#### Computational aspects

The simulation study is performed using R version 4.3.1 (R Core Team, 2023) and the following R packages: the mvtnorm package (Version 1.2-3, Genz & Bretz, 2009) to generate data, the lm() function included in the stats package (Version 4.3.1, R Core Team, 2023) to fit the different models, the SimDesign package (Version 2.13, Chalmers & Adkins, 2020) to set up and run the simulation study, and the ggplot2 package (Version 3.4.4, Wickham, 2016) to create visualizations. We executed the simulation study on a system running Ubuntu 22.04.4 LTS. A sessionInfo output with more information on the computational environment, a Dockerfile to reproduce it, and code and data to reproduce the study and its analysis are available at the Open Science Framework (https://osf.io/dfgvu/).

### Results

**Figure 4:**
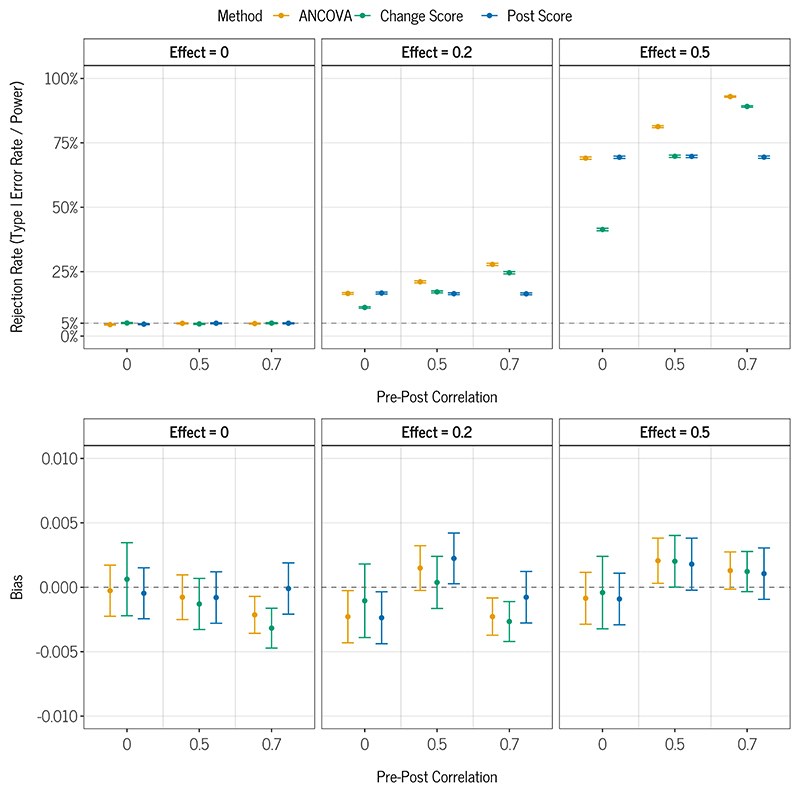
Estimated rejection rate (Power / Type I Error depending on DGM) and Bias of ANCOVA, Change Score Analysis and Post Score Analysis. *Note*. Error bars correspond to *±*1 Monte Carlo standard error. The y-axis in the bias plot is scaled only from −0.01 to 0.01, meaning that the bias can be considered negligible.

Figure 4 shows the results of the simulation study visually, Table 6 in the Appendix shows the same results numerically. No missing/non-convergent values were observed. We see from the Effect = 0 panel/rows that all methods maintain a Type I error rate close to 5% irrespective of the correlation between the pre–post measurements. For non-zero effects, when the pre–post measurement correlation is zero, ANCOVA and post-score analysis exhibit similar levels of power and both surpass change-score analysis. However, when the pre–post measure correlation increases to 0.7, change-score analysis shows higher power than post-score analysis, yet ANCOVA shows higher power than both other methods.

The lower panels in Figure 4 show the estimated bias of the methods. We see that all methods had essentially equivalent bias across all simulation conditions. Furthermore, the bias of all methods in all conditions was close to zero and, given the very small MCSEs, can be considered as negligible.

In sum, under the investigated scenarios, all methods produced unbiased effect estimates while ANCOVA consistently showed the highest power among the three methods. In almost all conditions, the Type I error rate was within one MCSE of the nominal rate of 5%, and all differences to the nominal rate were smaller than 0.5%. For this simple setting and the methods under study, there is a substantial amount of statistical theory that explains and predicts our results (see, e.g., Senn, 2008), which is not often the case for simulation studies. Our findings are also in line with previous simulation studies (Vickers, 2001).

## Discussion and recommendations

Our review of 100 articles published in prominent journals for methodological research in psychology shows considerable room for improvement in the design and reporting of simulation studies. The precision and uncertainty of estimated results are often neglected, as evidenced by the lack of justification for the number of simulation repetitions and the limited reporting of Monte Carlo uncertainty in most studies. The unavailability of openly accessible code and detailed information on the computational environment in most studies is a major barrier to critical evaluation, reproducibility, and incremental progress in methodological research. At the same time, we have also highlighted several positive examples from the literature that stood out for their transparent reporting or clear justification of the number of repetitions. In our opinion, these positive examples illustrate how simulation studies ought to be conducted and reported. Based on the insights from our literature review, we provide recommendations for researchers to improve the quality of simulation studies in the following (see Table 5, for an overview).

The quality of simulation studies can benefit from standardized design and reporting, such as with the ADEMP structure (Morris et al., 2019) that we have reviewed in this article. A standardized structure ensures that researchers think about important issues when designing their study and that all important information is reported. However, comprehensive reporting of what was done is not enough; researchers should also provide a rationale for the choices made in the design and analysis of their simulation study (e.g., justifications for data-generating mechanisms and analysis methods), as this is essential for readers and reviewers to assess the quality of evidence provided by the study.

As with any empirical study, it is important to acknowledge the uncertainty of the results within each simulation study, for example, by reporting Monte Carlo standard errors for estimated performance measures. Any observed pattern should only be interpreted if the associated Monte Carlo uncertainty (e.g., MCSE) is sufficiently small relative to the magnitude of the performance measure of interest. In order for researchers to draw meaningful conclusions, the uncertainty should already be taken into account in the design of the study, for example, by choosing the number of repetitions such that a sufficiently small Monte Carlo standard error is achieved. The formulae in Table 3 can be used for this purpose. The choice of the desired MCSE, and hence the number of repetitions required, is embedded in the trade-off between the generalizability and the precision of a simulation study. Researchers aiming for high precision in their performance measure estimates will usually be able to study fewer conditions, restricting the scope of their investigation and limiting the external validity and generalizability of their results. Therefore, setting the number of simulation repetitions too high can also waste computational resources that could be better spent investigating additional settings. Choosing the number of simulation repetitions to achieve desired precision, as explained in our article, can help researchers to make informed choices in this trade-off. However, even when simulation studies are carefully designed in advance, their scope is often narrow compared to all possible realistic settings. Researchers should avoid discrepancies between the scope of their simulation and the generality with which their results are reported.

Preregistration of a study protocol helps to make a transparent distinction between knowledge, decisions, and evaluation criteria that were present before or after the results were observed. At the same time, preregistration does not mean that the researcher’s hands are tied and that modifications to the study cannot be made, but rather that they should be transparently disclosed through amendments to the protocol. Fortunately, the issue of “double-dipping” on the same data to formulate and test hypotheses is less of a problem in simulation studies as new data can typically be generated cheaply (with certain exceptions, such as bootstrap or Monte Carlo methods). Rather, the purpose of preregistered protocols is to guide the planning of rigorous simulation studies, to provide other researchers with a transparent picture of the research process, and potentially receive peer feedback independent of the results. This concerns especially the selection of methods, data-generating mechanisms, conditions, and performance measures, which are often highly flexible in simulation studies. Researchers can also obtain feedback on their protocols from other researchers, especially if the protocol is publicly available (see, e.g., Kipruto & Sauerbrei, 2022). Moreover, fixing the criteria for the evaluation of the results a priori protects researchers from cognitive biases in the interpretation of results, such as hindsight bias, confirmation bias, or allegiance bias, that can blur their interpretation of simulation study results. Our ADEMP-PreReg template (https://zenodo.org/doi/10.5281/zenodo.10057883, development version: https://github.com/bsiepe/ADEMP-PreReg) can be used for preparing a (possibly preregistered) simulation study protocol, as a blueprint for the structured reporting of a simulation study, or as guidance document when reviewing a simulation study. In future work, this may be extended to a standardized reporting checklist created by a panel of experts on simulation studies, similar to risk-of-bias assessment tools for randomized controlled trials (Sterne et al., 2019) or reporting guidelines for prediction models in health care (Collins, Reitsma, Altman, & Moons, 2015).

To foster computational reproducibility and enable other researchers to build on a simulation study, we strongly recommend to share code, data, and other supplementary material. We recommend to upload files to a research data repository that accords with the FAIR principles (Wilkinson et al., 2016), such as OSF or Zenodo, as we encountered various dead links in our review, even from journals. Zenodo, in particular, offers great integration with GitHub which facilitates developing simulation code on GitHub (using the git version control system) and then archiving time-stamped versions or snapshots of the repository in a FAIR way on Zenodo with one click. Moreover, we recommend to report software versions and the computational environment used to run the study in detail. For example, for R users (the vast majority of researchers based on our review), we recommend to at least report the output of sessionInfo() in the supplementary material or code repository as a low-effort step for reporting necessary software versions and the computational environment. Ideally, data files containing the full output of a simulation study should be shared if possible.

Besides researchers conducting simulation studies themselves, other academic stakeholders can help raise the standards of methodological research. For example, during the peer-review process, reviewers and editors can encourage proper design and reporting of simulation studies, for instance, by guiding authors to justify the number of repetitions or to report Monte Carlo standard errors. Similarly, journals can promote higher standards for simulation studies by requiring authors to share code and/or data for articles that include simulation studies. This seems appropriate since conclusions from simulation studies heavily depend on the validity of their underlying code, and since there are usually no ethical concerns with publishing code and simulated data (with the exception of studies with data generating mechanisms based on resampling, where sharing the resampled data could be problematic). Mandatory code and data sharing, along with reproducibility checks and reproducibility badges, have already been adopted by several journals, for example, *Meta-Psychology* or *Biometrical Journal* which both have dedicated reproducibility teams that (partially) rerun simulation studies of submitted articles (Hofner, Schmid, & Edler, 2015; Lindsay, 2023). In a similar vein, journals could have specific calls for the replication and/or generalization of influential simulation studies (Giordano & Waller, 2020; Lohmann, Astivia, Morris, & Groenwold, 2022; Luijken et al., 2023).

## Conclusions

Simulation studies are a remarkably powerful tool. They allow methodologists to study the behavior of methods in virtually any situation they can imagine, often less limited by ethical, resource, or time constraints than most other types of research. At the same time, large-scale simulation studies can involve a considerable amount of costly human labor and energy-intensive computational resources. To reduce the waste of valuable resources, simulation studies should thus be planned and executed with care. Even more important, with the potentially long-lasting impact of simulation studies on scientific practice comes great responsibility to recognize the inherent uncertainty and the limited generalizability of their results. After all, simulation studies are experiments, and their success depends on the same factors as any other type of experiment—careful experimental design, consideration of sampling uncertainty, neutral evaluation of results, transparent reporting, and sharing of data and code so that other researchers can build on them.

While a more standardized approach to planning and reporting simulation studies can improve their overall quality, there are potential pitfalls. As with other types of empirical research, methodological research exists on a spectrum from exploratory to confirmatory, and different “phases” of research require different degrees of rigor and standardization but also generate different degrees of evidence (Heinze et al., 2024). An overly legalistic approach to standardization, such as requiring pre-registration even from exploratory early-stage studies, may therefore be unreasonable and potentially slow the field’s progress. Furthermore, while the ADEMP framework is broadly applicable to typical simulation studies in psychology, it is not a “one-size-fits-all” solution and adaptation to specific settings may be required, providing an opportunity for future research. Finally, while preregistration can help to control researcher degrees of freedom and post hoc interpretation of results, verifying the preregistration date is difficult, and researchers could potentially fake the date and gain unwarranted trust from others in the scientific community. There are some potential solutions, such as adversarial collaboration or time-stamping simulation runs on a cluster computer/server, but these need to be explored in more depth in future research.

Over the past two decades, psychology has proven to be a remarkably adaptive discipline, as exemplified by the widespread adoption of preregistration as well as open data, code, and materials (Munafò et al., 2017). We believe, therefore, that the time is ripe for a similar shift in methodological research toward more rigor and transparency in simulation studies.

## Supplementary Material

Appendix

## Figures and Tables

**Table 1:** Summary of the ADEMP Planning and Reporting Structure for Simulation Studies.

Step	Explanation	Example
**A**ims	What is the aim of the study?	To evaluate the hypothesis testing and estimation characteristics of different methods for analyzing pre–post measurements
**D**ata-generating mechanism	How are data sets generated?	Pre–post measurements are simulated from a bivariate normal distribution for two groups, with varying treatment effects and pre–post correlations
**E**stimands and other targets	What are the estimands and/or other targets of the study?	The null hypothesis of no effect between groups is the primary target, the treatment effect is the secondary estimand of interest
**M**ethods	Which methods are evaluated?	ANCOVA, change-score analysis, and post-score analysis
**P**erformance measures	Which performance measures are used?	Type I error rate, power, and bias

**Table 2:** Different Types of Statistical Tasks, their Target(s), and Typical Performance Measures.

Statistical Task	Target(s)	Typical performance measures
Estimation	Estimand(s) (True effects/parameters)	Bias, variance, mean square error, coverage, CI width
Hypothesis testing	True hypothesis(es)	Type I error rate, power
Model selection	True model(s)	Model-recovery rate, sensitivity, specificity
Prediction	New data	Prediction error, calibration, discrimination
Clustering	True cluster(s)	Fidelity to the true cluster structure
Design	Design characteristics	Expected sample size, minimum/maximum sample size, power, precision (for a fixed sample size)

*Note*. Table adapted from Morris et al. (2019, Table 3).

**Table 3:** *Definitions of Common Performance Measures, their Estimates, Monte Carlo Standard Errors (MCSE), and Number of Simulation Repetitions n*_sim_ *to Achieve a Desired* MCSE*.

Performance measure	Definition	Estimate	MCSE	*n*_sim_
Bias	$\text{E}(\hat{\theta })-\theta$	$\left(\sum_{i=1}^{n_{\text{sim}}}{\hat{\theta }}_{i}/n_{\text{sim}}\right)-\theta$	$\sqrt{S_{\hat{\theta }}^{2}/n_{\text{sim}}}$	$S_{\hat{\theta }}^{2}/{\text{MCSE}}_{*}^{2}$
Relative bias	$\{\text{E}(\hat{\theta })-\theta \}/\theta$	$\left\{\left(\sum_{i=1}^{n_{\text{sim }}}{\hat{\theta }}_{i}/n_{\text{sim }}\right)-\theta \right\}/\theta$	$\sqrt{S_{\hat{\theta }}^{2}/\left(\theta^{2}n_{\text{sim}}\right)}$	$S_{\hat{\theta }}^{2}/\left({\text{MCSE}}_{*}^{2}\theta^{2}\right)$
Mean square error (MSE)	$\text{E}\left\{{\left(\hat{\theta }-\theta \right)}^{2}\right\}$	$\sum_{i=1}^{n_{\text{sim}}}{\left({\hat{\theta }}_{i}-\theta \right)}^{2}/n_{\text{sim}}$	$\sqrt{S_{{\left(\hat{\theta }-\theta \right)}^{2}}^{2}/n_{\text{sim}}}$	$S_{{\left(\hat{\theta }-\theta \right)}^{2}}^{2}/{\text{MCSE}}_{*}^{2}$
Root mean square error (RMSE)	$\sqrt{\text{E}\left\{{\left(\hat{\theta }-\theta \right)}^{2}\right\}}$	$\sqrt{\sum_{i=1}^{n_{\text{sim}}}{\left({\hat{\theta }}_{i}-\theta \right)}^{2}/n_{\text{sim}}}$	$\sqrt{S_{{\left(\hat{\theta }-\theta \right)}^{2}}^{2}/\left(4n_{\text{sim}}\hat{\text{MSE}}\right)}$	$S_{{\left(\hat{\theta }-\theta \right)}^{2}}^{2}/\left(4\hat{\text{MSE}}{\text{ MCSE}}_{*}^{2}\right)$
Empirical variance	$\text{Var}(\hat{\theta })$	$S_{\hat{\theta }}^{2}$	$S_{\hat{\theta }}^{2}\sqrt{2/\left(n_{\text{sim}}-1\right)}$	$1+2{\left(S_{\hat{\theta }}^{2}\right)}^{2}/{\text{MCSE}}_{*}^{2}$
Empirical standard error	$\sqrt{\text{Var}(\hat{\theta })}$	$\sqrt{S_{\hat{\theta }}^{2}}$	$\sqrt{S_{\hat{\theta }}^{2}/\left\{2\left(n_{\text{sim}}-1\right)\right\}}$	$1+S_{\hat{\theta }}^{2}/\left(2{\text{MCSE}}_{*}^{2}\right)$
Coverage	Pr(CI includes *θ*)	$\sum_{i=1}^{n_{\text{sim }}}𝟙\left({\text{CI}}_{i}\;\text{includes}\;\theta \right)/n_{\text{sim }}$	$\sqrt{\hat{\text{Cov}}(1-\hat{\text{Cov}})/n_{\text{sim}}}$	$\hat{\text{Cov}}(1-\hat{\text{Cov}})/{\text{MCSE}}_{*}^{2}$
Power (or Type I error rate)	Pr(Test rejects *H*_0_)	$\sum_{i=1}^{n_{\text{sim}\;}}𝟙({\text{Test}}_{i}\;\text{rejects }H_{0})/n_{\text{sim }}$	$\sqrt{\hat{\text{ Pow }}(1-\hat{\text{ Pow }})/n_{\text{sim }}}$	$\hat{\text{Pow}}(1-\hat{\text{Pow}})/{\text{MCSE}}_{*}^{2}$
Mean CI width	E(CI_upper_ − CI_lower_)	$\sum_{i=1}^{n_{\text{sim }}}\left(\text{C}{\text{I}}_{i,\text{upper}\;}-\text{C}{\text{I}}_{i,\text{lower}}\right)/n_{\text{sim }}$	$\sqrt{S_{W}^{2}/n_{\text{sim }}}$	$S_{W}^{2}/{\text{MCSE}}_{*}^{2}$
Mean of generic statistic *G*	E(*G*)	$\sum_{i=1}^{n_{\text{sim}}}G_{i}/n_{\text{sim}}$	$\sqrt{S_{G}^{2}/n_{\text{sim}}}$	$S_{G}^{2}/{\text{MCSE}}_{*}^{2}$

*Note*. Table adapted from Table 6 in Morris et al. (2019) E(*X*) and Var(*X*) are the expected value and variance of a random variable *X*, respectively. Summation is denoted by $\sum_{i=1}^{n}\;x_{i}=x_{1}+x_{2}+\cdots +x_{n-1}+x_{n}$. $\hat{\theta }$ is an estimator of the estimand *θ*, and ${\hat{\theta }}_{i}$ is the estimate obtained from simulation *i* 𝟙 (CI_*i*_ includes *θ*) and 𝟙 (Test_*i*_ rejects *H*_0_) are 1 if the respective event occurred in simulation *i* and 0 otherwise $\hat{\text{MSE}}\text{, }\hat{\text{Cov}}$, and $\hat{\text{Pow}}$ denote the estimated MSE, coverage, and power, respectively. MCSE_*_ denotes the desired MCSE when calculating the number of repetitions *n*_sim_. The sample variance of the estimates is $S_{\hat{\theta }}^{2}=\sum_{i=1}^{n_{\text{sim }}}{\{{\hat{\theta }}_{i}-\left(\sum_{i=1}^{n_{\text{sim }}}{\hat{\theta }}_{i}/n_{\text{sim }}\right)\}}^{2}/\left(n_{\text{sim }}-1\right)$ The sample variance of the square errors is $S_{{\left(\hat{\theta }-\theta \right)}^{2}}^{2}=\sum_{i=1}^{n_{\text{sim}}}{\left[{\left({\hat{\theta }}_{i}-\theta \right)}^{2}-\left\{\sum_{i=1}^{n_{\text{sim}}}{\left({\hat{\theta }}_{i}-\theta \right)}^{2}/n_{\text{sim}}\right\}\right]}^{2}/\left(n_{\text{sim}}-1\right)$ The sample variance of the CI widths is $S_{W}^{2}=\sum_{i=1}^{n_{\text{sim}}}{[\left(\text{C}{\text{I}}_{i,\text{upper}\;}-\text{C}{\text{I}}_{i,\text{lower}\;}\right)-\left\{\sum_{i=1}^{n_{\text{sim}}}\left(\text{C}{\text{I}}_{i,\text{upper }}-\text{C}{\text{I}}_{i,\text{lower}\;}\right)/n_{\text{sim }}\right\}]}^{2}/\left(n_{\text{sim }}-1\right)$ The sample variance of a generic statistic *G* is $S_{G}^{2}=\sum_{i=1}^{n_{\text{sim}}}{\{G_{i}-\left(\sum_{i=1}^{n_{\text{sim}}}G_{i}/n_{\text{sim}}\right)\}}^{2}/\left(n_{\text{sim}}-1\right)$ with *G*_*i*_ the statistic obtained from simulation *i*. For example, *G* may be a measure of predictive performance.

**Table 4:** Summary of Common Pitfalls Identified in the Literature Review.

Step	Pitfalls
Aims	Not reporting specific aims
Data-generating mechanism	Not summarizing simulation conditions and data-generating mechanism in a structured way (e.g., bullet points, tables)
	Not providing justification and Monte Carlo uncertainty coupled with a small number of simulation repetitions
Estimands and other targets	Not defining estimands / targets clearly, especially in models with many parameters
Methods	Not clearly listing all of the compared methods and their specifications
Performance measures	Not clearly defining performance measures
	Not clearly defining how performance measures are aggregated
	Not reporting Monte Carlo uncertainty
	Not reporting convergence
Computational aspects	Not reporting computational environment (operating system, software, and package versions)
	Not using persistent repositories for sharing code and data (e.g., publisher or university repositories)
	Not sharing code and data

*Note*. Pitfalls were not all coded explicitly, but summarized from the quantitative results of the literature review and discussions between the reviewing authors.

**Table 5:** Recommendations for Methodological Research Using Simulation Studies.

Recommendation
1. Provide a rationale for all relevant choices in design and analysis (e.g., justifications for data-generating mechanism conditions and analysis methods)
2. Use a standardized structure for planning and reporting of simulation studies (e.g., ADEMP)
3. Report Monte Carlo uncertainty (e.g., Monte Carlo standard errors, uncertainty visualizations)
4. Choose the number of simulation repetitions to achieve desired precision
5. Write (and possibly preregister) study protocol to guide simulation design and to disclose the state of knowledge, prior expectations, and evaluation criteria before seeing the results (e.g., using the ADEMP-PreReg template)
6. Avoid selective reporting of results that lead to desired outcomes
7. Acknowledge the limited generalizability of a single simulation study
8. Report software versions and environment (e.g., using sessionInfo() in R)
9. Upload code, data, results, and other supplements to a FAIR research data repository (e.g., OSF or Zenodo)
10. Journals/Editors/Reviewers: Promote higher reporting standards and open code/data (e.g., require code/data sharing)

## Footnotes

1 This may initially sound confusing to the reader, as some might expect the target of the simulation would be the outcome—Type I error rate of the hypothesis test. However, this would be the *performance measure* that indicates the methods’ performance for the given *target*—the null hypothesis.

2 See the documentation of the gtExtras **R** package (Mock, 2024) for examples.

3 Random number streams are available in R through different packages, e.g., parallel (R Core Team, 2023), rstream (Leydold, 2022), or doRNG (Gaujoux, 2023).

4 Due to an oversight, we did not review the last issue of BRM in 2022 but rather continued with the first 2021 issues of the journals.

5 We used version 0.1.0 of the template in this example as archived at https://doi.org/10.5281/zenodo.10057884.

6 An alternative way of writing this model is lm(I(post – pre) ∼ treatment)
