# Supplementary material for: Simulation Studies for Methodological Research in Psychology: A Standardized Template for Planning, Preregistration, and Reporting
Source: Psychol Methods. Author manuscript; Available in PMC 2024 Nov 22. (PMC7616844; doi:10.1037/met0000695)
Supplement: Appendix [file EMS200172-supplement-Appendix.pdf]

White, I. R., Pham, T. M., Quartagno, M., & Morris, T. P. (2023). How to check a simulation study. *International Journal of Epidemiology*. doi: 10.1093/ije/dyad134

Wickham, H. (2016). *ggplot2: Elegant graphics for data analysis*. Springer-Verlag New York. Retrieved 2023-10-17, from https://ggplot2.tidyverse.org

Wilkinson, M. D., Dumontier, M., Aalbersberg, I. J., Appleton, G., Axton, M., Baak, A., . . . Mons, B. (2016). The FAIR guiding principles for scientific data management and stewardship. *Scientific Data*, *3*(160018). doi: 10.1038/sdata.2016.18

Appendix

A summary of inter-rater agreement for the studies that were most difficult to code is provided in Figure 5. The wording of the questions and their answer options are available in the preregistration of our review (https://osf.io/8cbfd). Overall, the agreement between raters appears to be acceptable. Low agreement is especially pronounced in questions about the estimands, where the ambiguity of reporting combined with the complexity of some models often made the assessment of a specific number of estimands very difficult. As we used studies that we identified as most difficult for our assessment of agreement, we consider the proportions found here as a lower bound for the overall agreement across all studies. Also, higher rates of disagreement in some questions here again indicate the need for more clarity in the reporting of studies. In the case of disagreement, we kept the rating of the initial reviewer for the analyses in the manuscript.

Numerical results of the example simulation study on methods for the analysis of pre–post measurements are given in Table 6.

Figure 5

Agreement between Raters

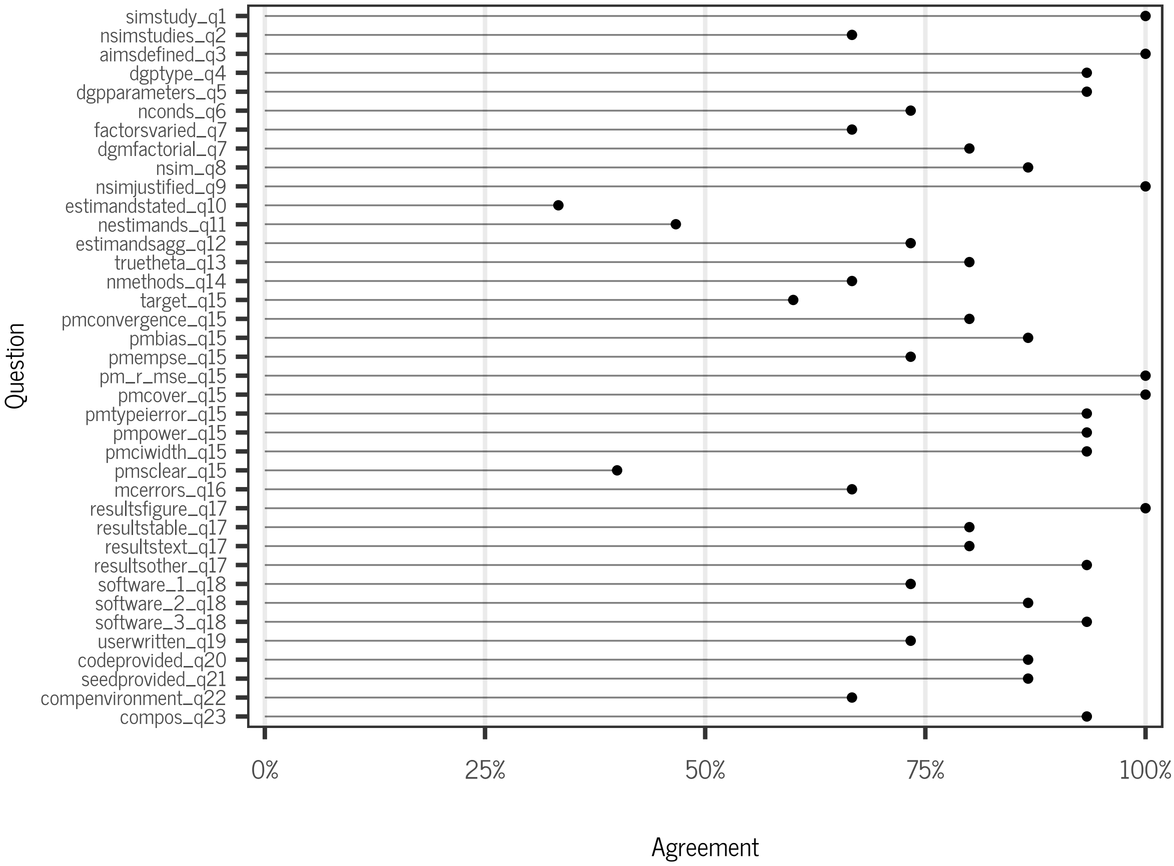

Note. Proportion of agreement between the three raters for 15 papers with a low or medium confidence rating. Two studies that were also rated for agreement were not assessed here, as the raters chose different simulation studies therein.

Table 6

Estimated Power / Type I Error Rate (MCSE) and Bias (MCSE) of ANCOVA, Change Score Analysis, and Post Score Analysis Across Simulation Conditions

Condition		Power / Type I Error Rate			Bias		
Correlation	Effect	ANCOVA	Change score	Post score	ANCOVA	Change score	Post score
0.00	0.00	0.0447 (0.0021)	0.0508 (0.0022)	0.0464 (0.0021)	−0.0003 (0.0020)	0.0006 (0.0028)	−0.0005 (0.0020)
0.00	0.20	0.1655 (0.0037)	0.1111 (0.0031)	0.1671 (0.0037)	−0.0023 (0.0020)	−0.0010 (0.0029)	−0.0024 (0.0020)
0.00	0.50	0.6907 (0.0046)	0.4137 (0.0049)	0.6940 (0.0046)	−0.0009 (0.0020)	−0.0004 (0.0028)	−0.0009 (0.0020)
0.50	0.00	0.0496 (0.0022)	0.0474 (0.0021)	0.0500 (0.0022)	−0.0008 (0.0017)	−0.0013 (0.0020)	−0.0008 (0.0020)
0.50	0.20	0.2108 (0.0041)	0.1715 (0.0038)	0.1646 (0.0037)	0.0015 (0.0017)	0.0004 (0.0020)	0.0022 (0.0020)
0.50	0.50	0.8130 (0.0039)	0.6978 (0.0046)	0.6973 (0.0046)	0.0021 (0.0018)	0.0020 (0.0020)	0.0018 (0.0020)
0.70	0.00	0.0487 (0.0022)	0.0503 (0.0022)	0.0500 (0.0022)	−0.0021 (0.0014)	−0.0032 (0.0015)	−0.0001 (0.0020)
0.70	0.20	0.2782 (0.0045)	0.2459 (0.0043)	0.1641 (0.0037)	−0.0023 (0.0014)	−0.0027 (0.0016)	−0.0008 (0.0020)
0.70	0.50	0.9296 (0.0026)	0.8913 (0.0031)	0.6944 (0.0046)	0.0013 (0.0014)	0.0012 (0.0016)	0.0011 (0.0020)
